# *Toxoplasma* infection in sheep from south of Iran monitored by serological and molecular methods; risk assessment to meat consumers

**DOI:** 10.14202/vetworld.2016.850-855

**Published:** 2016-08-13

**Authors:** Belal Armand, Kavous Solhjoo, Manoochehr Shabani-Kordshooli, Mohammad Hasan Davami, Mehdi Sadeghi

**Affiliations:** 1 Department of Parasitology and Mycology, Jahrom University of Medical Sciences, Jahrom, Iran; 2 Department of Parasitic Disease, Zoonoses Research Center, Jahrom University of Medical Sciences, Jahrom, Iran; 3 Department of Biology, Islamic Azad University, Shiraz Branch, Shiraz, Iran

**Keywords:** *B1* gene, enzyme-linked immunosorbent assay, meat consumers, nested-polymerase chain reaction, sheep, *Toxoplasma gondii*

## Abstract

**Aim:**

*Toxoplasma gondii* has a clinical and veterinary importance as it is known to cause congenital disease and abortion both in humans and livestock. Since the contaminated lamb is one of the sources of human infection, this study was performed to determine the prevalence of *T. gondii* in sheep in south of Iran.

**Materials and Methods:**

Sera and tissue samples (diaphragm and heart) were collected from 370 sheep from slaughterhouse of Jahrom. The samples were taken from both sexes and from 6 to 60 months age. Specific immunoglobulin G antibodies to *T. gondii* were examined with enzyme-linked immunosorbent assay, and *B1* gene nested-polymerase chain reaction detection was done to survey the tissue samples.

**Results:**

The total prevalence of *Toxoplasma* infection among sheep was found to be 35.94% and 34.32% based on serological and molecular method, respectively. According to serologic and molecular findings, the females were more positive than males for *Toxoplasma*; maximum frequency of positive samples was observed in 24-36 months and the positive samples had been collected more in spring than in summer, but no statistical correlation was observed between prevalence rate and the age and sex of animals or season of sampling.

**Conclusion:**

*T. gondii* is widely distributed in sheep in Jahrom with a rate comparable with other parts of Iran and the world. It suggested a widespread exposure of sheep in this region to *T. gondii*. Thus, consumption of undercooked or raw meat presents the transmission risk of the parasite and this might be considered as an important public health problem, mainly for high-risk groups such as the pregnant and the immunodeficient.

## Introduction

Zoonotic diseases are one of the major public health problems in many countries. One of these diseases is toxoplasmosis. Toxoplasmosis is a widespread zoonosis caused by *Toxoplasma gondii*, a ubiquitous coccidian parasite of felines, man and many wild or domestic warm-blooded animals [1]. Herbivores acquire infection generally by the ingestion of oocysts, shed by infected cats, in water or contaminated food, and humans become infected post-natally by ingesting tissue cysts from undercooked meat [2].

The parasite has clinical and veterinary importance as it is known to cause congenital disease and abortion both in humans and livestock [3]. Acquired toxoplasmosis is normally asymptomatic or has mild non-specific symptoms in immunocompetent persons, while in immunocompromised individuals might be life threatening [4].

It is well known that meat from persistently infected animals is one of the most important potential sources of human toxoplasmosis [5]. Hence, it is necessary to investigate the prevalence of *T. gondii* infection among meat producing animals. Sheep are important to the economy of many countries because they are a source of nutrition for humans. Epidemiological investigations have revealed a significant correlation between human toxoplasmosis and the consumption of raw or undercooked meat or its products [2].

*T. gondii* in sheep is a source of infection for humans and carnivorous animals [6]. Various serological and molecular tests have been widely used by researchers in epidemiological studies on animal and human toxoplasmosis worldwide [7-12]. The prevalence of toxoplasmosis was reported in different livestock such as sheep in different parts of Iran which is varied between 13.8% and 35% for sheep [13-15]. In addition, a seroprevalence rate of 51.8% has been reported for all parts of Iran [16]. The seroprevalence rate of *Toxoplasma* infection in Fars province, by focusing on Shiraz city, has been reported to be 26.5% in sheep [17]. Moreover, tissue cysts were observed in 38% of tissue samples of sheep by molecular methods in southwest of Iran [18].

There is little information concerning toxoplasmosis rate in sheep in southern parts of Iran. Furthermore, sheep breeding is significantly common in this area, and since the contaminated lamb is one of the sources of human infection, this study was performed to determine the prevalence of *T. gondii* in sheep using both serological and molecular methods in the south of Fars. This survey provides an accurate picture of the risk of exposure to *T. gondii* in a common source of meat products.

## Materials and Methods

### Ethical approval

The experiment on animals including all procedures of this study was approved by the local Ethical Committee in Jahrom University of Medical Sciences.

### Study area and sampling

The animals were chosen from the main slaughterhouse of Jahrom district, south of Fars province, where animals gathered from different regions of the district, between Aprils and June 2013. Jahrom is situated in a zone with 1050 m height from sea level where the temperature can become high in summer and a mild winter. Within the study area, 370 sheep blood samples were randomly collected from slaughtered sheep. In addition, the tissue samples were taken from diaphragm and heart of all animals for molecular examination. The animals had been born and raised in the region and were intended for human consumption. Demographic information such as sex, age, and breeding area of samples was recorded. The age of animals was ranging from 6 to 60 months.

### Serologic examination

Sera of samples were separated and stored at −20°C until assayed. Specific immunoglobulin G antibodies to *T. gondii* were examined with enzyme-linked immune assay using *T. gondii* Human Kit of DIA. PRO Italian Company and Abcam Company sheep serum conjugate. Negative control was obtained from a newborn sheep. After the study protocol had been approved by the Local Ethical Committee, the sheep was infected by two steps injection of live tachyzoites intramuscular and subdermal for obtaining positive control sample.

The optical density (OD) was read with a spectrophotometer (MULTISKAN MCC/340 P VERSION 2.33) at 492 nm. The absorbance average of each serum tested in duplicate was divided by the cut off (mean absorbance of negative serum samples plus three standard deviations) to determine the reactivity index (RI). Serum with RI 1 was considered positive.

### Molecular examination

For extraction of DNA, the tissue samples were homogenized and DNA was extracted using phenol-chloroform and Proteinase K. The extracted DNA was stored at −20°C until use. Two polymerase chain reaction (PCR) primer pairs of the *B1* gene were used has been showed on Table-1. These primers amplifying a 193 bp bond at the initial phase and a 96 bp fragment at the second round of nested-PCR.

**Table-1:** Position and sequences of the primer pairs used in nestedPCR.

Oligonucleotide primer	Sequence	Sequence position
Outer primer (sense strand)	5’GGAACTGCATCCGTTCATGAG3’	694-714
Outer primer (nonsense strand)	5’TCTTTAAAGCGTTCGTGGTC3’	887-868
Inner primer (sense strand)	5’TGCATAGGTTGCAGTCACTG3’	757-776
Inner primer (nonsense strand)	5’GGCGACCAATCTGCGAATACACC3’	853-831

PCR: Polymerase chain reaction

The first amplification was performed in 20 µl of PCR-PreMix (Bioneer Company, South Korea) reaction mixture and 1 µl of each primer and 2.5 µl of extracted DNA (5-50 nanogram). The PCR condition was 93°C for 10 min, followed by 40 cycles of 93°C for 10 s of denaturation, 57°C for 10 s of annealing and 72°C for 30 s of extension, and the last extension step at 72°C for 5 min.

The second amplification was carried out in the same volumes as the first reaction with the 1 µl of the first round product as template and 1 µl of each inner primer. The PCR condition was 93°C for 10 min, followed by 40 cycles of 93°C for 10 s of denaturation, 62.5°C for 10 s of annealing and 72°C for 15 s of extension, and the last extension step at 72°C for 5 min.

Each amplification run contained two negative controls (doubly distilled water and negative control of DNA extraction) and one positive control (DNA extracted from RH *T. gondii* tachyzoite). The PCR products were analyzed by electrophoresis in a 2% agarose gel stained with ethidium bromide (0.5 mg/ml). The DNA fragments were visualized under ultraviolet illumination.

### Statistical analysis

The Chi-square test was used to clarify whether sex, age, or season of sampling was associated with the prevalence rate of *T. gondii* in sheep. The results were analyzed by SPSS software (version 13) and a p<0.05 was considered as significant positive correlation.

## Results

The number of 370 sheep (279 female and 91 male) were examined using both serological and molecular methods. The sampling was done during two seasons of spring (193 sheep) and summer (177 sheep). The animals were categorized in five age groups including 0-12 months (122), 12-24 months (70), 24-36 months (125), 36-48 months (34), and 48-60 months (19).

### Serological findings

In this study, the samples with higher than 170 OD were considered positive. Anti-*Toxoplasma* antibodies were detected in sera of 133 out of 370 (35.94%) animals. Considering the sex of animals, however the females (100 cases) were more seropositive than males (33 cases) for *Toxoplasma*, but the differences were not statistically significant (p=0.942). Moreover, the maximum and minimum frequency of positive samples were observed in 24-36 months (42.40%) and 48-60 months (31.58%) age groups, respectively, however no significant correlation was found between age and seropositivity to toxoplasmosis (p=0.470). In addition, 76 positive samples had been taken in spring and 57 in summer, but statistical analysis was indicated no correlation between season and the seropositivity (p=0.151). Serological findings are shown in Table-2 in detail.

**Table-2:** Distribution of *T. gondii* infection in sheep based on serological and molecular results in correlation to sex, age group and season of sampling.

Category	Number of animal examined	Number of ELISA positive (%)	Number of nested-PCR positive (%)
Season			
Spring	193	76 (39.37)	82 (42.48)
Summer	177	57 (32.20)	45 (25.42)
Sex			
Male	91	33 (36.26)	31 (34.06)
Female	279	100 (35.84)	96 (34.40)
Age group			
0-12	122	39 (31.96)	25 (20.49)
12-24	70	23 (32.85)	25 (35.71)
24-36	125	53 (42.40)	57 (45.60)
36-48	34	12 (35.29)	13 (38.23)
48-60	19	6 (31.58)	7 (36.84)
Total	370	133 (35.94)	127 (34.32)

*T. gondii: Toxoplasma gondii*, ELISA: Enzyme-linked immunosorbent assay, PCR: Polymerase chain reaction

### Molecular findings

**Figure-1:**
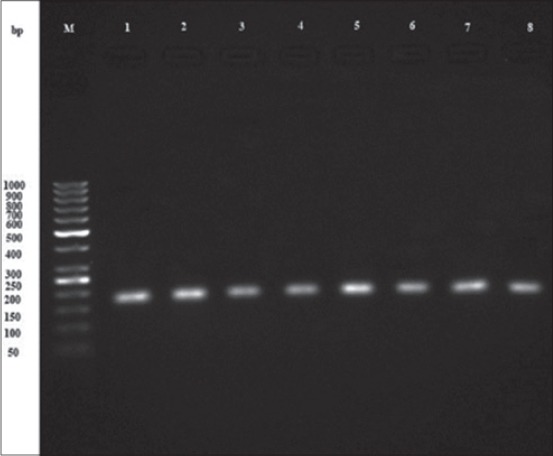
Electrophoresis result from primary step product of B1 gene nested-polymerase chain reaction products: M-100 bp marker, 1 - Positive control, 2-9: 193 bp bond of positive samples.

**Figure-2:**
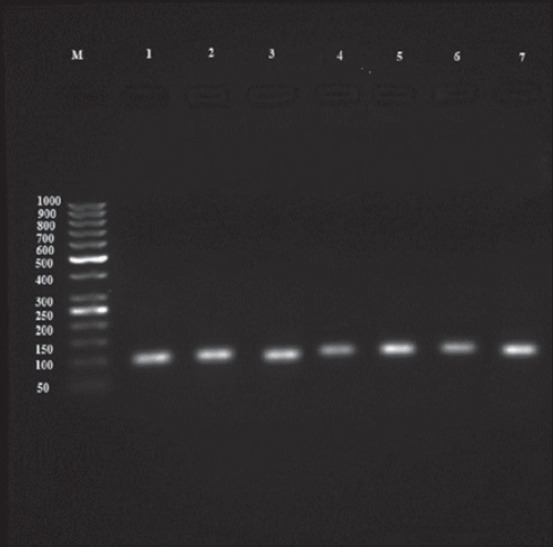
Electrophoresis result from secondary step product of B1 gene nested- Polymerase chain reaction: M-50 bp marker, 1- Positive control, 2-9: 96 bp bond of positive samples.

A nested-PCR assay was done for tissue (heart and diaphragm) samples taken from 370 sheep to amplify the *B1* gene. In general, 127 cases (34.32%) showed a 193 bp bond at initial step and a 96 bp bond at the second round of nested-PCR assay (Figures-1 and 2). All the positive samples had been identified positive based on serological results. The statistical difference between the prevalence rates of *Toxoplasma* infection among females (96 cases) and males (31 cases) was not significant (p=0.886). Furthermore, no positive correlation was found between the age of animals and the rate of infection, in which the highest and lowest prevalence rate were observed in 3 years old age group and 1 year old age group, respectively (p=0.055). Although 82 cases of positive samples had been collected in spring and 45 of them had been taken in summer, there was no positive relation observed between the rate of prevalence and season of sampling (p=0.138). Molecular findings are shown in Table-2 in detail.

## Discussion

Nowadays, toxoplasmosis is considered as one of the most important food-borne diseases. Humans are getting the disease by consuming infected meat of livestock including sheep [19]. The results of this study showed that 35.94% of sheep have anti *T. gondii* antibody in their sera. In addition, 34.32% of animals’ tissue contains *T. gondii* tissue cyst. Toxoplasmosis prevalence rate in the various zones of the world is variable, with ranging from 0% to 100% in different countries [20,21]. The differences observed could be due to diverse husbandry practices, lifestyles of the residents, traditions and the climatic variations from one region to another, which are known as essential elements in epidemiological investigations [22].

Our result is one of the highest rates which have been reported so far in different parts of Iran. Our findings were significantly more than those who reported in Kerman 3.3% seropositivity in sheep [23]. This could be due to the differences of weather in the two areas. Kerman is a desert zone, while Jahrom with the vast citrus gardens looks more like a forest zone rather than a poor average annual rainfall area. In other aspect, cats and other felids play an important role in preserving and spreading of *T. gondii* in hosts such as livestock (e.g., sheep) because they are considered as the major source of the oocysts that contaminate the environment and after sporulation become infectious to man and animals [22]. Humidity and temperate condition favor the oocyst survival. To that effect, Fayer has indicated that the toxoplasmosis prevalence rate is elevated in humid and hot zones compared to dry regions and this gap is more probably due to the high viability of the oocysts in these environments [24].

In addition, it seems that the prevalence of *T. gondii* in sheep in the Jahrom district was slightly more than other parts of Iran such as Uroomye [25], Shahre-Kord [26], and Kurdistan [27] provinces, in the west and the mountainous part of Iran. In the mentioned regions, there were reports of the seropositivity of 21.1%, 29.1%, and 21.74%, respectively. Moreover, the average seroprevalence in Iran which was reported by Hashemi [28] about 24.5% is distinctly lower than our finding in this area. In comparison with other parts of Fars province, again our results showed a little difference, in which Asgari observed that 29.5% of sheep in northern part of Fars province hold anti-*Toxoplasma* antibody in the sera [29].

More interestingly, Sharifi reported 35% sheep toxoplasmosis in three different regions, north of Iran, which is the most humid and forest part of the country. That was clearly similar to the findings of the present study. Sharifi, also, observed that seropositivity was higher in the western parts of the province where animals had been exposed to an environment contaminated with greater numbers of *T. gondii* oocysts, as a result of differences in the levels of humidity in these three areas [14]. Other studies across the world have confirmed this fact. In this regard, van der Puije in his study has indicated that the sheep toxoplasmosis varies from 20% in a dry region to 39% in forest areas [30]. It is worth mentioning that our findings were distinctly less than Greece, a Mediterranean country, where 48.6% of sheep were categorized seropositive [31]. Other studies in Ghana [30] and Portugal [32] which showed the same seroprevalence range as our study, around 33.2% and 33.6% infection, respectively.

To the best of this study, all the samples were subjected to a molecular survey to clarify the existence of parasite in the animals’ tissue. The major purpose of this step was to determine whether *T. gondii* was present as a contaminant of human foodstuffs, and if so, the level of contamination present. Our findings show a high rate of toxoplasmosis infection in sheep tissues. Detecting this high level of contaminant in a small part of animal tissue (5 g) poses an alarming public health risk because it means that considerable numbers of farm animals currently contributing to the food chain of people carry *T. gondii*.

Our finding demonstrates the presence of *T. gondii* DNA within the samples, regardless of the viability of parasites which might initiate a human infection. However, it seems highly probable that a significant portion of the parasites observed in the present study would have been viable. Because, it is well proved that *T. gondii* tissue cysts are considerably strong and remaining viable for weeks at temperatures of 1-4°C, and temperatures above 67°C or below −12°C are needed to see a significant loss of viability [33,34].

In other aspects, consuming the barbequed meat (kebab) and the processed meat products such as sausages have been dramatically increased in the recent years; which it clearly represents a significant risk of infection.

The results of this study showed that 33 out of 91 samples of male and 100 out of 279 samples of female animals were seropositive, but there was no statistical correlation between gender of sheep and the rate of seropositivity. This was in contrary to those who found a significant correlation between ewes and lambs [35]; whereas it was similar to the studies done in Iran and other parts of the world [26,29,31]. Some studies indicated a connection between the age of animals and the rate of *Toxoplasma* infection [11,14]; however, such connection was not observed in the current study like *Asgari* who did not find a statistical correlation in this kind [29]. In general, the prevalence of *T. gondii* varies with the methods of testing and cut-off values. It seems that the sensitivity and specificity of different tests are different that they might have some effects on results [36].

## Conclusion

Regarding the foregone discussion, *T. gondii* is widely distributed in sheep in this region with a rate comparable with other parts of Iran and world. Obviously, the consumption of their undercooked or raw meat presents a risk of transmission of the parasite. This might be considered as an important public health problem, mainly for high-risk groups such as the pregnant and the immunodeficient. Likewise, other meats from other kinds of animals are currently used in Jahrom. It seems highly important to identify the other probable sources of infection to have a better vision to the role of meat diet in human infection with *T. gondii* and also to define the zoonotic aspect of this parasite.

## Authors’ Contributions

BA and KS have designed the concept and supervised the plan of work and also have prepared the manuscript. MS and MSK have contributed in sample collection, administrative, technical, and material support. KS and MHD have analyzed and interpreted the data. All authors have read and approved the final manuscript.
